# Investigation of the underlying causes of malnutrition among children in the Nyabihu and Ngororero districts of Rwanda: a cross-sectional descriptive survey design

**DOI:** 10.11604/pamj.2025.52.121.48967

**Published:** 2025-11-21

**Authors:** Francois Niyongabo Niyonzima, Aloys Iyamuremye, Ezechiel Nsabayezu, Xavier Cheseto, Emmanuel Rangiryayo, Furaha Umutoni Alida, Umuhoza Karemera Noella Josiane, Patrick Gad Iradukunda, Kevin Ndayisaba, Marc Antoine Ndisanze

**Affiliations:** 1 Department of Math and Science, University of Rwanda, College of Education, Kigali, Rwanda,; 2 Department of Chemistry, Jomo Kenyatta University of Agriculture and Technology, Nairobi, Kenya,; 3 College of Agriculture, Forestry and Food Science, School of Agriculture and Food Science, University of Rwanda, Musanze, Rwanda,; 4 College of Arts and Social Sciences, University of Rwanda, Center for Conflict Management, Kigali, Rwanda,; 5 Repolicy Research Centre, Kigali, Rwanda,; 6 Volcano Green Invest Ltd, Musanze, Rwanda,; 7 Department of Biotechnologies, Institut d'Enseignement Supérieur de Ruhengeri (INES), Musanze, Rwanda

**Keywords:** Child malnutrition, dietary diversity, health access, food insecurity

## Abstract

**Introduction:**

malnutrition remains a critical public health challenge in Rwanda, particularly in rural districts like Nyabihu and Ngororero, where children face chronic undernutrition despite ongoing national interventions. This study investigates the underlying causes of malnutrition among children in Nyabihu and Ngororero districts.

**Methods:**

using a structured questionnaire, data were gathered from 46 parents to assess household income, nutrition knowledge, dietary practices, sanitation, and access to health services. Independent t-tests, MANOVA, and Chi-square tests were utilized to examine relationships between variables.

**Results:**

most households earn less than 50,000 RWF per month, reflecting widespread poverty. Significant gender-based disparities were observed in engagement with community malnutrition programs, with women (M= 0.96, SD= 0.19) demonstrating significantly higher involvement than males (M= 0.58, SD= 0.42), t (46) = 2.45, p= 0.017. Despite general awareness of principles of balanced diets, this knowledge is not translating into practice. Most households reported poor dietary diversity and inadequate intake of proteins, dairy, fruits, and vegetables, leading to deficiencies in essential nutrients. Alarmingly, a large proportion of children are not benefiting from available nutrition interventions. Perceptions of malnutrition varied significantly by gender (p= 0.013) and occupation (p= 0.036), though not by age (p= 0.119).

**Conclusion:**

the study highlights the urgent need for targeted, multisectoral strategies that address both knowledge-practice gaps and systemic barriers. In addition to improving food availability, integrated interventions focusing on maternal education, dietary diversity, hygiene, and equitable access to healthcare services are crucial for achieving sustainable improvements in child nutrition.

## Introduction

Malnutrition remains among the most pressing public health challenges in sub-Saharan Africa, with severe implications for child development, cognitive performance, and long-term national productivity [1-3]. In this context, inadequate food intake, poor breastfeeding practices, micronutrient deficiencies, and recurring infections continue to undermine child health and survival. In addition, factors such as low maternal education, cultural food taboos, limited access to diverse foods, poor sanitation, and gender inequality further exacerbate the burden of undernutrition [4-6]. The consequences of malnutrition are far-reaching, including increased susceptibility to anemia, respiratory infections, weakened immunity, and, ultimately, impaired physical and cognitive development [7,8]. While community health workers play a key role in identifying and managing malnourished children in Rwanda, barriers such as long distances to health facilities, delayed care-seeking behavior, financial hardship, and low awareness of symptoms contribute to delayed treatment and worsened outcomes [9]. In rural districts like Nyabihu and Ngororero, many households lack access to clean water, adequate sanitation, and nearby health facilities, and have limited infrastructure [9,10]. While a balanced diet containing carbohydrates, proteins, vitamins, minerals, and fats is essential for proper growth and development, food choices in rural Rwandan households are often driven by availability rather than nutritional value [11,12]. Diets in these regions are predominantly composed of starchy staples such as cassava, maize, sweet potatoes, and Irish potatoes, with minimal intake of protein-rich and micronutrient-dense foods such as fruits, vegetables, dairy, and animal-source products [13-15]. Consequently, deficiencies in key nutrients such as vitamin A, iron, and zinc are common and contribute significantly to growth faltering and weakened immune systems in children. Although Rwanda has achieved high national immunization coverage, gaps remain in rural communities due to long travel distances, vaccine stockouts, misinformation, and weak follow-up systems [5]. Strengthening caregiver engagement and community health systems is essential to closing these gaps.

In some rural areas, misunderstandings persist regarding the causes of malnutrition. Some caregivers attribute these conditions to hereditary or spiritual causes, undermining early intervention efforts. Continuous community sensitization is therefore critical for improving recognition of early signs, promoting appropriate feeding practices, and addressing cultural misconceptions [14,16]. While the Rwandan government and its partners have made commendable strides in addressing malnutrition, including nutrition education and food supplementation for vulnerable families, challenges around coverage, sustainability, and community ownership remain [13]. In Ngororero and Nyabihu districts, malnutrition affects approximately 33% of children under four years [17]. Typical diets in these communities consist of low-cost, high-calorie meals such as porridge, boiled roots or tubers, and occasionally beans or peas, offering insufficient protein, fats, and essential vitamins [18]. These dietary inadequacies, combined with socio-economic hardship, contribute to a high number of malnourished children [19]. Despite ongoing national efforts to reduce child malnutrition, elevated rates persist in remote areas such as Nyabihu and Ngororero. Preliminary data point to a complex interplay of behavioral, cultural, and socioeconomic factors [20]. However, the specific root causes in these districts have not been comprehensively explored. This study aims to fill the gap by exploring the underlying socioeconomic, behavioral, and systemic causes of childhood malnutrition in Nyabihu and Ngororero. Therefore, the following are specific research objectives: i) to identify the socioeconomic factors contributing to child malnutrition in Nyabihu and Ngororero districts; ii) to examine household dietary practices, food access, and nutritional knowledge among parents and caregivers; iii) to assess the role of sanitation, hygiene, and health service access in influencing child nutritional outcomes; iv) to analyze community participation in nutrition-related programs and their impact on child health.

## Methods

**Study design:** a cross-sectional descriptive survey design was utilized to collect both qualitative and quantitative data. The study was conducted from November 2024 to May 2025 and explored household-level factors such as socioeconomic status, nutritional practices, and health conditions among families affected by malnutrition for children aged 24-48 months.

**Study setting:** the study was conducted in Ngororero and Nyabihu districts, located in the Western Province of Rwanda. These districts were purposively selected because they have persistently high prevalence rates of child malnutrition (approximately 33%), and face structural challenges such as poverty, food insecurity, and limited access to healthcare services [21-23]. Additionally, health centers in these districts also report frequent malnutrition cases, making them representative and relevant sites for exploring the underlying causes of child malnutrition [24].

**Study participants:** the study population consisted of children aged 24-48 months who were clinically identified as undernourished. However, a total of 46 parents participated in the study (18 from Ngororero and 28 from Nyabihu districts). The sample size was determined based on the number of eligible and consenting parents present at the purposively selected highly affected health centers during the data collection period. Because the health centers manage a consistently high number of malnutrition cases, this approach ensured adequate representation of affected households within the study districts.

**Data collection instrument:** data collection took place in May 2025 and was carried out by trained members of the project team. A structured questionnaire was administered using the Kobo Toolbox platform [25]. The questionnaire comprised sections covering demographic characteristics, sanitation and hygiene, nutrition awareness and practices, health service access, socioeconomic conditions, and dietary habits and food diversity.

**Data analysis:** quantitative data were analyzed using Excel 2016 and SPSS version 25. To address objective 1 (socio-economic factor), descriptive statistics (means, percentages, and frequencies) were used to summarize household income, occupation, and demographic variables. For objective 2 (dietary practices and nutrition knowledge), cross-tabulations and Chi-square tests examined associations between knowledge of balanced diets and actual feeding practices. To address objective 3 (sanitation, hygiene, and health service access), descriptive statistics and Chi-square tests assessed access levels and their relationship with children´s nutritional outcomes. Finally, for objective 4 (community participation in nutrition-related programs), independent t-tests compared male and female participation, while MANOVA was used to determine statistical associations between variables, with a significance level set at p < 0.05. Qualitative responses obtained from open-ended questions were analyzed thematically. Recurring themes related to feeding practices, healthcare barriers, and socio-cultural factors were identified and categorized accordingly.

## Results

**Demographic information of participants:** the demographic characteristics of the study participants are summarized in Table 1. All households had at least one child aged between 2 and 4 years, representing a total of 46 children included in the study.

**Table 1:** demographic characteristics of study participants, recruited from the Nyabihu and Ngororero districts, Rwanda, from November 2024 to May 2025 (N=46)

	Demography	Number of participants
**Gender**	Male	4
	Female	42
	Total	46
**Age**	Under 18 years	1
	Between 18-25 years	6
	Between 26-35 years	21
	Between 36-45 years	14
	46 and above	4
	**Total**	46
**Marital status**	Single	5
	Married	28
	Divorced	8
	Widow	5
	Total	46
**Number of children**	1 Child	11
	2 Children	8
	3 Children	10
	4 Children	7
	5 and above Children	10
	**Total**	46
Age of your children	2-4 years	46
	Total	46

**General living conditions of participants:** a large majority (74%) are engaged in farming or livestock activities. Part-time work accounts for 20% of the participants, while only 4% are self-employed. A very small number (2%) work as government or private sector employees. A significant majority (93%) of the participants earn below 50,000 RWF per month. A smaller portion, 4%, earn between 50,000 and 100,000 RWF, while only 2% have a monthly income ranging from 100,000 to 200,000 RWF.

**Figure 1:**
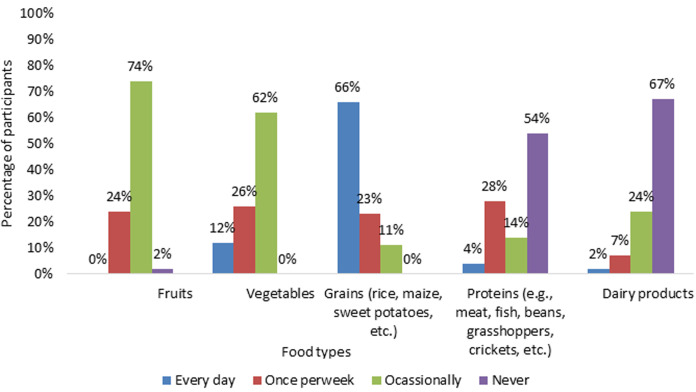
pattern in the consumption of various food types among parents of malnourished children aged 24-48 months in Nyabihu and Ngororero districts, Rwanda, November 2024 to May 2025 (N=46)

**Trends in the consumption of various food types:** Figure 1 illustrates pattern in the consumption of various food types among parents of malnourished children aged 24-48 months in Nyabihu and Ngororero districts, Rwanda, November 2024 to May 2025 (N=46). The findings showed that most participants consume more grains and an inadequate intake of nutrient-rich foods. The participants were also asked whether they had access to all foods mentioned in Figure 1. They responded that 69.6% have no access, while 30.4% have access. They obtained food from the market, accounting for 74% of the total. Meanwhile, only 26% are produced or sourced from their farm.

**Assessment of respondents´ awareness of balanced diet principles for children:** 72% of respondents have received training or education on balanced diets for children, and 78% report knowing what constitutes a balanced diet for children. Despite this high level of awareness, only 22% believe their children are receiving a balanced diet, while 78% think otherwise. Multivariate analysis of variance (MANOVA) results revealed p-values for all factors that are greater than the conventional significance level of 0.05, indicating that there are no statistically significant differences in awareness of balanced diet principles based on these demographic variables (Table 2).

**Table 2:** multivariate analysis of respondents’ awareness of balanced diet principles for children by gender, age, and occupation among parents of malnourished children in Nyabihu and Ngororero districts, Rwanda (N=46)

Multivariate Tests						
**Effect**		**Value**	**F**	**Hypothesis df**	**Error df**	**Sig**.
Factor	Wilks' Lambda	947	1.045b	2.000	37.000	.362
Gender	Wilks' Lambda	922	1.572b	2.000	37.000	.221
Age	Wilks' Lambda	863	710b	8.000	74.000	.682
Job	Wilks' Lambda	926	728b	4.000	74.000	.576

b. Exact statistic

**Assessment of respondents´ awareness of balanced diet principles for children:** 72% of respondents have received training or education on balanced diets for children, and 78% report knowing what constitutes a balanced diet for children. Despite this high level of awareness, only 22% believe their children are receiving a balanced diet, while 78% think otherwise. Multivariate analysis of variance (MANOVA) results revealed p-values for all factors that are greater than the conventional significance level of 0.05, indicating that there are no statistically significant differences in awareness of balanced diet principles based on these demographic variables (Table 2).

**Prevalence of health complications among malnourished children:** diarrhea is the most common issue (47%), followed by vomiting (20%), frequent infections (19%, primarily respiratory/skin), and fever (14%). This pattern demonstrates how malnutrition and illness create a vicious cycle of nutritional deficiencies.

**Analysis of child treatment patterns for malnutrition:** eighty percent (80%) of respondents reported that their child had received treatment for malnutrition, while 20% stated their child had not received any form of treatment or intervention. Three main forms to treat malnutrition were identified: flour (46%), pills (43%), and milk (11%).

**Analysis of typical daily meals consumed by children:** forty percent (40%) of children eat only one meal per day, 47% consume two meals, and 14% are able to eat three meals daily. The fact that 87% of children are eating fewer than three meals per day points to food insecurity and economic hardship in many households. In terms of food taken by children, such as protein foods rich in vitamins and minerals, only 21% consume these foods, 58% occasionally, and 21% don’t have access to these foods. A significant majority of respondents (72.22%) indicated that their children consume such food only a few times a week. A smaller proportion (16.67%) reported rare consumption, which may reflect the underlying food insecurity. Only 5.56% of the participants reported that their children consume nutritional foods daily, and an equally small percentage reported weekly consumption.

**Challenges faced by caregivers in ensuring a balanced diet for their children:** the most commonly cited challenge was a lack of money to buy diverse foods (61%). Limited knowledge on nutrition was the second most reported barrier (26%). Lack of access to nutritious foods accounted for 11% of responses. Only 1% of respondents cited a lack of time to prepare meals or cultural/traditional beliefs as significant challenges.

**Access to water, hygiene, and sanitation:** seventy-seven (77%) have access to clean and safe water for drinking, while 9% sometimes have access to clean and safe water. However, 14% of them have no access to clean and safe water for drinking. In addition, 57% of respondents use public taps or community boreholes, 7% rely on rivers or lakes, and only 36% have access to a borehole. In terms of hygiene at home, 80% of households reported having access to a latrine or toilet, while 20% do not. Secondly, when it comes to sanitation practices, such as using latrines and washing hands regularly, only 59% of respondents affirmed that they should practice proper sanitation, while a substantial 41% admitted they do not.

**Health and medical care access:** eighty-nine (89%) of children have regular access to healthcare services, while 11% do not. However, 11% of those without regular access remain at risk of missing routine immunizations, nutrition screenings, and timely treatment. Regarding distance, the distances to health facilities vary: 37% live within 1-3 km, 55% within 1-5 km, and 20% live more than 6 km away.

**Analysis of child vaccination status based on national guidelines:** ninety-eight (98%) of children were reported to have received vaccinations according to the national immunization schedule. Additionally, 96% of children had received deworming treatment in the past six months.

**Knowledge and awareness of malnutrition:** forty-eight (48%) of respondents demonstrated an understanding of the main causes. However, 23% of respondents explicitly stated they did not know the causes, and a notable 30% were unsure, reflecting a significant knowledge gap. The study aims to determine whether there is a statistically significant difference in participants´ knowledge and awareness about malnutrition by gender, age, and job. In this context, the chi-square was used. The results indicate that respondents´ understanding of the main causes of malnutrition in children varies significantly by gender and job type (p<0.05), while age differences were not statistically significant (p>0.05). In this regard, females demonstrated a higher level of understanding compared to males (Table 3). Additionally, civil servants were significantly more likely to understand the causes compared to those in farming/livestock or self-employment.

**Table 3:** Chi-square analysis of participants’ knowledge of malnutrition by gender, age, and occupation among parents of malnourished children in Nyabihu and Ngororero districts, Rwanda, November 2024 to May 2025 (N=46)

Variable	Test Used	Chi-square (χ^2^)	df	p-value	Interpretation
Gender	Chi-square test	4.25	2	0.036	Statistically significant (p < 0.05)
Age group	Chi-square test	16.45	8	0.119	Not statistically significant (p>0.05)
Job type	Chi-square test	12.75	4	0.013	Statistically significant (p < 0.05)

**Causes of malnutrition among children:** seventy-three (73%) identified a lack of food as the primary cause. Other factors mentioned include poor diet or inadequate nutrition (12%), lack of breastfeeding (7%), frequent illness or infections (5%), and poor hygiene or sanitation (3%).

**Community-based programs or services that address malnutrition:** eighty-nine (89%) were involved in community-based programs, while 11% were not participating. An independent samples t-test was utilized to compare levels of involvement in the malnutrition program between male and female participants. Results revealed a statistically significant difference in mean involvement scores, with females (M= 0.96, SD= 0.19) showing significantly higher involvement than males (M= 0.58, SD= 0.42), t (46) = 2.45, p= 0.017. The qualitative responses indicate that participants are actively involved in the community-based malnutrition program, primarily through activities conducted at the health center and within their villages via the “*Irerero*” through “*Gikuriro*” initiative.

**Access to external nutritional support from local organizations and the government:** in terms of support received, 57% of households reported receiving external support, while the remaining 43% received no support. Specifically, they reported receiving items such as milk and *Sosoma* flour from health centers, as well as additional support from the *Gikuriro* program within their villages. Participants emphasized that addressing malnutrition requires a multi-pronged approach: providing balanced diets, especially for vulnerable families; offering financial or material support; and enhancing parental education through training and awareness programs. However, common challenges such as poverty, limited access to diverse food items, and a lack of nutritional knowledge were recurrent.

## Discussion

**Socioeconomic and demographic constraints:** the investigation revealed significant socioeconomic and demographic constraints that contribute to malnutrition in Nyabihu and Ngororero districts. The socioeconomic challenges, such as low income and poverty, are among the most pressing issues contributing to child malnutrition in these districts. Additionally, limited financial resources can also be attributed to malnutrition. This gap between awareness and affordability highlights a major structural barrier to improving child nutrition [5]. Additionally, large family sizes and limited employment opportunities exacerbate the economic strain, leading to food insecurity and inadequate care for children [26]. These findings highlight the urgent need for economic empowerment initiatives and poverty reduction strategies in rural areas.

**Limited dietary diversity and food insecurity:** the findings illustrate that many parents strive to care for their children, but they still face significant challenges, particularly a lack of a balanced and diverse diet. They rely on eating grains like maize or cassava, but rarely eat protein-rich foods like meat, milk, beans, or eggs. This situation is common in many rural African communities [27]. However, this kind of diet does not give children the vitamins and minerals they need to grow well and stay healthy. Poor diets like this can cause “hidden hunger,” where children may eat enough food but still suffer from serious nutrient deficiencies [27-29]. This shows a big gap between what people know and what they are able to practice. This problem has been found in other studies as well. For example, Blum *et al*. found that even when parents know the right foods to give their children, they often cannot afford them or cannot find them easily [30]. Thus, just giving information is not enough. Families need support to turn their knowledge into action. Another important issue found in the study is that many children eat only one or two meals per day, and even those meals often lack important nutrients. This was supported by Chambers *et al*. who highlighted that many rural households in Africa face this problem, especially during dry seasons or when harvests are poor [31]. Although ready-to-use food supplements can help to treat malnutrition quickly [32], they do not solve the long-term problem. To end malnutrition, families need to be able to grow or buy nutritious food themselves, without always relying on aid.

**Health status and access to care:** the study results indicated high levels of child malnutrition, with 86% of children being underweight. The study also showed that many malnourished children are getting sick frequently, especially with diarrhea, vomiting, infections, and fever. This is a serious concern. It is well known that poor nutrition weakens the immune system, making it easier for children to get sick [33]. At the same time, when children have infections, their bodies don´t absorb food properly, which worsens malnutrition. Limited access to health care due to distance or affordability constraints compounded these problems, creating a vicious cycle of illness and malnutrition [34]. This back-and-forth relationship between illness and poor nutrition has been explained in many studies [7,35,36]. It means that any effort to fight malnutrition must also improve sanitation and health care.

**Knowledge gaps and behavior change needs:** the study revealed that most of the participants are aware of balanced diet principles. However, few of them were able to provide such diets to their children. The lack of time, gender dynamics, and cultural beliefs may contribute to this discrepancy. This gap between practice and knowledge may be attributed to poverty, inadequate behavioral change support, and limited food access. Similarly, Oduro *et al*. reported that the inability to implement hygiene practices and good feeding may be ascribed to poverty, lack of support programs, and/or cultural beliefs in rural areas [37]. This matches with UNICEF´s model on child nutrition, which explains that poor income, food insecurity, and lack of services are the main causes of malnutrition [26]. Another important finding is that knowledge about the causes of malnutrition varied by gender and job type. In this sense, women and civil servants were more informed than men and those working in farming. This Azizi Fard *et al*. corroborates, who found that formal education and job exposure help people understand health and nutrition better [38]. The need for behavior change strategies and community education that are designed by gender and occupation is necessary [39].

**Structural barriers and programmatic gaps:** the study highlights structural barriers such as food markets, dependence on aid-based nutrition interventions, inadequate sanitation practices, and limited access to clean water. Thus, even though most families had access to clean water and toilets, many caregivers still said they don´t practice good hygiene, such as washing hands or using toilets regularly. This is a missed opportunity. Studies show that poor hygiene increases the risk of diarrhea and other infections, which can make malnutrition worse [39,40]. Thus, education on hygiene and behavior change is still needed. One encouraging result was that most parents were actively involved in community nutrition programs, like *Gikuriro* and *Irerero*. These programs provide nutrition education, flour, milk, and other support. According to Rousseau *et al*. such community-based programs work best when they are close to the people and address local needs [41]. Programs like these are important because they give families practical tools and ongoing support to improve nutrition. Studies in other African countries [42] also show that community programs, combined with health services, can help reduce child malnutrition. Importantly, women were the most active in such programs, reinforcing the importance of supporting female caregivers while also involving men in nutrition and childcare initiatives. These gaps highlight the need for inclusive, multisectoral programs addressing income, gender equity, education, and food systems [43].

**Limitations:** this study was limited by its small sample size (n= 46), which may restrict generalizability. Moreover, reliance on self-reported data may introduce recall or social desirability bias. Nonetheless, the richness of both quantitative and qualitative data provides valuable insights into the lived realities of nutrition-insecure households.

## Conclusion

This study examined the current situation of child malnutrition in Nyabihu and Ngororero districts and revealed serious challenges that families face in providing adequate nutrition for their children. While many parents have knowledge about balanced diets and show a willingness to participate in nutrition-related programs, there remains a significant gap between knowledge and practice. Poor dietary diversity, food insecurity, and low income are major barriers. Most children eat only one or two meals a day, and the meals often lack essential nutrients such as proteins, vitamins, and minerals. In addition, frequent illnesses like diarrhea and infections worsen the condition of malnourished children. Even though access to health services, vaccinations, and deworming is relatively high, these alone are not enough to address malnutrition. The study also found that community-based programs such as *Gikuriro* and *Irerero* play an important role in supporting families, particularly mothers. However, not all families benefit equally from these programs, and male involvement remains low. Integrated, gender-sensitive, and community-based strategies are urgently needed to improve nutritional outcomes and ensure every child’s right to healthy development in rural Rwanda.

### What is known about this topic

- In Ngororero and Nyabihu districts, malnutrition affects approximately 33% of children under five years;
- Typical diets in these communities consist of low-cost, high-calorie meals such as porridge, boiled roots or tubers, and occasionally beans or peas, offering insufficient protein, fats, and essential vitamins;
- Preliminary data point to a complex interplay of behavioral, cultural, and socioeconomic factors; however, the specific root causes in these districts have not been comprehensively explored.

### What this study adds

- This study reveals a gap between parental awareness of balanced diets and the practical ability to implement them;
- It identifies differences in awareness of malnutrition causes across gender and occupation groups;
- And it also emphasizes the interconnected role of socioeconomic, cultural, and systemic factors in shaping child nutrition.
